# Genetic evidence for an origin of the Armenians from Bronze Age mixing of multiple populations

**DOI:** 10.1038/ejhg.2015.206

**Published:** 2015-10-21

**Authors:** Marc Haber, Massimo Mezzavilla, Yali Xue, David Comas, Paolo Gasparini, Pierre Zalloua, Chris Tyler-Smith

**Affiliations:** 1 The Wellcome Trust Sanger Institute, Wellcome Trust Genome Campus, Hinxton, Cambridgeshire, UK; 2 Institute for Maternal and Child Health -IRCCS ‘Burlo Garofolo'—Trieste, University of Trieste, Trieste, Italy; 3 Institut de Biologia Evolutiva (CSIC–UPF), Departament de Ciències de la Salut i de la Vida, Universitat Pompeu Fabra, Barcelona, Spain; 4 The Lebanese American University, Chouran, Beirut, Lebanon

## Abstract

The Armenians are a culturally isolated population who historically inhabited a region in the Near East bounded by the Mediterranean and Black seas and the Caucasus, but remain under-represented in genetic studies and have a complex history including a major geographic displacement during World War I. Here, we analyse genome-wide variation in 173 Armenians and compare them with 78 other worldwide populations. We find that Armenians form a distinctive cluster linking the Near East, Europe, and the Caucasus. We show that Armenian diversity can be explained by several mixtures of Eurasian populations that occurred between ~3000 and ~2000 bce, a period characterized by major population migrations after the domestication of the horse, appearance of chariots, and the rise of advanced civilizations in the Near East. However, genetic signals of population mixture cease after ~1200 bce when Bronze Age civilizations in the Eastern Mediterranean world suddenly and violently collapsed. Armenians have since remained isolated and genetic structure within the population developed ~500 years ago when Armenia was divided between the Ottomans and the Safavid Empire in Iran. Finally, we show that Armenians have higher genetic affinity to Neolithic Europeans than other present-day Near Easterners, and that 29% of Armenian ancestry may originate from an ancestral population that is best represented by Neolithic Europeans.

## Introduction

Insights into the human past come from diverse areas including history, archaeology, linguistics, and, increasingly, genetics. The observed patterns of present-day genetic diversity can be compared with models that include past population processes such as migration, divergence and admixture, and the best model chosen. These models often require representing ancestral populations and mostly consider present-day populations as direct descendants of the ancient inhabitants of a region. However, archaeological and genetic data reveal that human history has often been shaped by regional or localized population movements that can confound simple demographic models.^1, 2^ Ancient DNA (aDNA) studies have also shown that the genetic landscape has been continuously shifting,^3, 4^ possibly triggered by environmental and cultural transitions. aDNA research is useful for understanding past demographic events; however, samples are limited and obtaining aDNA from warm climates remains a challenge. We have previously shown that studying genetic isolates also provides insights into human genetic variation and past demographic events.^5^ For example, by studying Jews, Druze, and Christians from the Near East, we showed that the region had more genetic affinity to Europe 2000 years ago than at present.^5^

**Figure 1:**
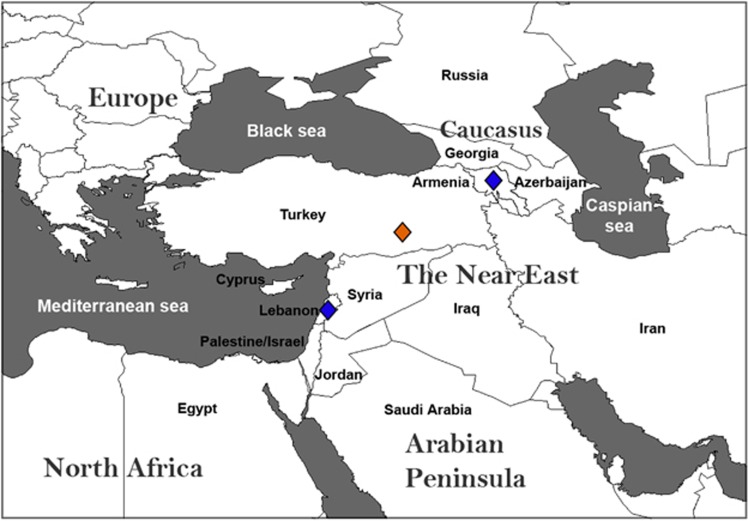
Map of the Near East and surrounding regions. The map shows the location of the present-day Armenia and neighbouring countries. Blue lozenges show the recruitment sites for the Armenian samples used in this study. Political turmoil during World War I resulted in the displacement of the East Turkey Armenian population (orange lozenge) to present-day Armenia or to several other nearby countries such as Lebanon.

In the present study, we investigate the Armenians, a population today confined to the Caucasus but who occupied Eastern Turkey, reaching as far as the Mediterranean coast, until the start of the twentieth century (CE; Figure 1). Political turmoil in the region during World War I resulted in the displacement of the Armenian population and its restriction today to an area in the Caucasus between the Black and the Caspian seas. Armenians are an ethno-linguistic-religious group distinct from their surrounding populations. They have their own church, the Armenian Apostolic Church, which was founded in the first CE and became in 301 CE the first branch of Christianity to be adopted as a state religion. They have also their own alphabet and language, which is classified as an independent branch of the Indo-European language family. The Armenian language is a subject of interest and debate among linguists for its distinctive phonological developments within Indo-European languages and for its affinity to Balkan languages such as Greek and Albanian. The historical homeland of the Armenians sits north of the Fertile Crescent, a region of substantial importance to modern human evolution. Genetic and archaeological data suggest that farmers expanding from this region during the Neolithic populated Europe and interacted/admixed with pre-existing hunter-gatherer populations.^6^ Furthermore, Armenia's location may have been important for the spread of Indo-European languages, since it is believed to encompass or be close to the Proto-Indo-European homeland (Anatolia or Pontic Steppe) from which the Indo-Europeans and their culture spread to Western Europe, Central Asia, and India.

Previous genetic studies of Armenians are scarce and genome-wide analysis is limited to a few Armenian samples in broad surveys without any detailed analysis. Armenians were found to have genetic affinity to several other populations including the Jews, Druze, and Lebanese Christians, in addition to showing genetic continuity with the Caucasus.^5, 7, 8^

In this study, we analyse newly generated genome-wide data from Armenians, as well as available data from 78 other worldwide populations. We investigate genetic signatures of past events such as the emergence of Armenians as an ethnic group, the cultural changes in the Near East, and the expansions of ancient populations in this region.

## Materials and methods

### Subjects and the genetic data sets

Armenian samples were collected from Lebanon (*n*=39) and Armenia: Chambarak (*n*=30), Dprabak (*n*=18), Gavar (*n*=12), Martuni (*n*=19), Yegvard (*n*=11), and Yerevan (*n*=9). Armenian individuals recruited in Lebanon traced their ancestry to East Turkey; they signed informed consent approved by the IRB of the Lebanese American University and were genotyped on Illumina 610 or 660 K bead arrays.

Armenian subjects recruited from present-day republic of Armenia signed consents approved by the ethical committee of the Maternal and Child Health Institute IRCCS-Burlo Garofolo Hospital (Trieste, Italy). Samples were genotyped on Illumina HumanOmniExpress and described previously by Mezzavilla *et al.*^9^

Genotype data can be downloaded as VCF files from the European Variation Archive www.ebi.ac.uk/eva under accession number PRJEB9822 or as plink files from ftp://ngs.sanger.ac.uk/scratch/project/team19/Armenian.

In addition, Armenian samples (*n*=35) were added along with 1509 samples from the literature that represent 78 worldwide populations.^5, 7, 8, 10^

PLINK^11^ was used for data management and quality control. The required genotyping success rate was set to 99%, sex-linked and mitochondrial SNPs removed, SNPs with MAF <0.001 and H–W *P*-value <0.000001 also removed, leaving 300 899 SNPs. Genotypes were phased with SHAPEIT^12^ using the 1000 Genomes Phase 1 haplotypes.^13^

### Population structure

Principal components were computed with EIGENSOFT v 4.2^14^ using 78 global populations, and the Armenian samples were projected onto the plot. The Bayesian information criterion (BIC) was computed by mclust (http://www.stat.washington.edu/mclust) over the first 10 principal components of the projected Armenian samples on the global PCA. The best model to classify the Armenians according to the BIC values was with three components (clusters; Supplementary Figure S1).

The inference of population relations from haplotypes was assessed using Chromopainter^15^ with 10 000 000 burn-in and runtime and 10 000 MCMC samples. A bifurcating tree of relationships among these populations was built using fineSTRUCTURE^15^ (Supplementary Figure S2). We investigated convergence by running fineSTRUCTURE twice with identical parameters but different random seeds and examined the pairwise coincidence matrix visually using the fineSTRUCTURE GUI. The two runs were identical, suggesting good convergence.

The effective population size of the Armenians was estimated from linkage disequilibrium (LD) and the time of divergence between the two major groups was calculated using *NeON*^16^ with default parameters. The function uses *Ne* and the genetic distance (*Fst*) between populations to estimate their time of divergence. *Fst* was calculated using the software 4P.^17^ The generation time used was 28 years.

### Admixture analysis

We used *f3* statistics^18^ *f3(A; B,C)*, where a significantly negative statistic provides evidence that *A* is derived from an admixture of populations related to *B* and *C*. We tested all possible *f3* statistics in our data set and calculated SE using blocks of 500 SNPs.^19^ To date the time of admixture, we used *ALDER*^20^, which computes the weighted LD statistic to make inferences about population admixture. The reference populations consisted of 1300 samples and 53 populations reduced from the original data set by removing populations that are themselves highly admixed (Supplementary Table 1). We collected results that were significant (*z*-score >|4|) and summarize the findings in Table 1 after pooling populations into respective geographical groups. Sardinians appear to have a distinctive admixture pattern from other West Europeans and are therefore shown separately. Sardinians have a European component but appear to have been less affected than other Europeans by the post-Neolithic demographic changes in Europe. Consequently, Sardinians retain high affinity to Neolithic European farmers such as the Tyrolean Iceman^21^ and samples from the Early Neolithic Körös culture.^22^

For tests of genetic affinity to Neolithic Europeans, we merged our samples with the genome of the Tyrolean Iceman.^21^ We downloaded the BAM file mapped to hg18 and called all variants using *GATK*.^23^ liftOver (http://genome.ucsc.edu) was used to convert the coordinates to hg19; the final data set consisted of 91 115 SNPs.

For tests of genetic affinity to Mesolithic Europeans, we merged our data set with the genome of the La Braña sample.^24^ We downloaded the BAM file mapped to hg19 and called the variants using *GATK.* The final data set consisted of 103 627 SNPs.

We applied *TreeMix*^19^, rooting the tree with a Denisovan genome, and estimated SEs using blocks of 500 SNPs. We generated 100 bootstrap replicates by resampling blocks of 500 SNPs to assess the stability of the tree topology. We used outgroup *f3* statistics^3, 18^ in the form of *f3(Yoruba; Iceman, X)* and *f3(Yoruba; La Braña, X)* to assess the shared genetic history of the ancient Europeans with the modern populations. In the absence of admixture with Yoruba, deviation from 0 will be a function of the shared genetic history of the ancient Europeans and the non-African population.

## Results

### Armenians' relationship to world populations

**Figure 2:**
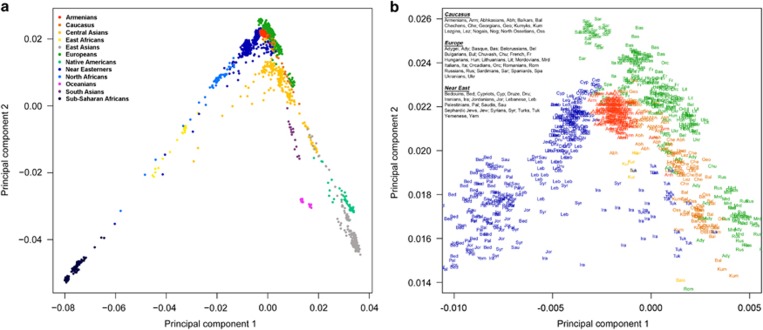
Principal component analysis of >240 000 SNPs showing the top two components. (**a**) The position of Armenians in a global genetic diversity sample based on 78 populations from 11 geographical regions. Armenians (173 individuals) were projected to the plot and therefore did not contribute to the observed global structure. (**b**) A magnification shows that the Armenians (red) demonstrate genetic continuity with the Near East, Europe, and the Caucasus.

To study the Armenians' genetic relationship to worldwide populations, we computed principal components using 78 populations (Supplementary Table 1) and projected the Armenians onto the plot in a procedure called ‘PCA projection'^14^ (Figure 2a), which ensures that the PCA patterns are not affected by the large number of Armenians used in the analysis. We observe that Armenians form a distinctive cluster bounded by Europeans, Near Easterners, and the Caucasus populations. More specifically, Armenians are close to (1) Spaniards, Italians, and Romanians from Europe; (2) Lebanese, Jews, Druze, and Cypriots from the Near East; and (3) Georgians and Abkhazians from the Caucasus (Figure 2b). The position of the Armenians within the global genetic diversity appears to mirror the geographical location of Turkey. Previous genetic studies have generally used Turks as representatives of ancient populations from Turkey. Our results show that Turks are genetically shifted towards Central Asians, a pattern consistent with a history of mixture with populations from this region.

These diversity patterns observed in the PCA motivated formal testing of admixture in Armenians and other regional populations.

### Admixture in the Near East

**Figure 3:**
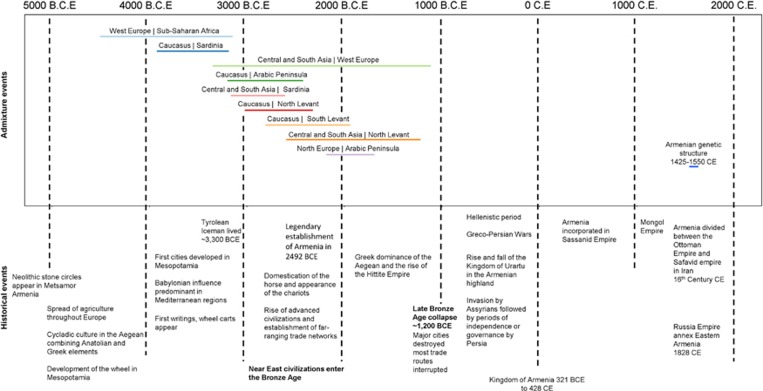
Genetically inferred source populations for Armenians, admixture times and genetic structure. Admixture events were estimated using decay of linkage disequilibrium with regional populations as sources for Armenians. Each horizontal coloured line indicates an admixture event and its width reflects the estimated date of admixture and SE. The plot also shows the estimated date of establishment of genetic structure within Armenians (1494–1545 CE). Major historical events and cultural developments in the Near East are shown at the bottom.

To formally test for population mixture in Armenians, we performed a *3-population* test^25^ in the form of *f3(Armenian; A, B),* where a significantly negative value of the *f3* statistic implies that Armenians descend from a mixture of the populations represented by *A* and *B*, chosen from the 78 global populations. We found signals of mixture from several African and Eurasian populations (Table 1, Figure 3). The most significantly negative *f3* statistics are from a mixture of populations related to Sardinians and Central Asians, followed by several mixtures of populations from the Caucasus, Arabian Peninsula, the Levant, Europe, and Africa. We sought to date these mixture of events using exponential decay of admixture-induced LD. The oldest mixture events appear to be between populations related to sub-Saharan Africans and West Europeans occurring ~3800 bce, followed closely by a mixture of Sardinian and Caucasus-related populations. Later, several mixture events occurred from 3000 to 1200 bce involving diverse Eurasian populations (Table 1, Figure 3).

We compared the patterns of admixture in Armenians with those of other regional populations and detected signals of recent admixture in most other populations. For example, we find 7.9% (±0.4) East Asian ancestry in Turks from admixture occurring 800 (±170) years ago. We also detect sub-Saharan African gene flow 850 (±85) years ago in Syrians, Palestinians and Jordanians.

### Structure of the Armenian population

To investigate the presence of genetic structure within the Armenian population, we performed model-based clustering on the values of the Armenian samples from the global PCA. The BIC computed by *MCLUST* suggests the best model to classify the Armenians is λ_k_*A* (diagonal distribution, variable volume, and equal shape) with three components (clusters). We observe the following: (1) Armenians in the diaspora that trace their origin to historical Western Armenia (modern-day East Turkey) form one group (Supplementary Figure S1, Cluster 1). (2) Armenians in modern-day Armenia (historical Eastern Armenia) are split into two major groups: 33% form Cluster 1 and 57% form Cluster 2 (Supplementary Figure S1). This structure could be the result of the Western Armenians' migration to the East after the events of 1915 CE that displaced the entire Western Armenian population. (3) A few Armenians recruited from Chambarak and Maykop (Republic of Adygea, Russia) form an outlier to the two major Armenian clusters (Supplementary Figure S1, Cluster 3).

We investigated Armenian structure further using a procedure called ‘chromosome painting',^15^ which reconstructs the haplotype of every individual (receiver) in a data set using the haplotypes of other individuals (donors) in the data set. We then constructed a tree that infers population relationships and similarities (Supplementary Figure S2). We found, similarly to our previous clustering results, a fine genetic structure that splits Armenians into two major groups that are more similar to each other than to any other global population. The node containing most Armenians is deep compared with many other nodes containing several diverse regional populations. This probably reflects a prolonged isolation of the Armenians from their surrounding populations as suggested by the LD-based admixture tests.

We estimate from the LD patterns that divergence between the two major Armenian groups started 450–575 years ago (Figure 3).

### Relationship to ancient Europeans

**Figure 4:**
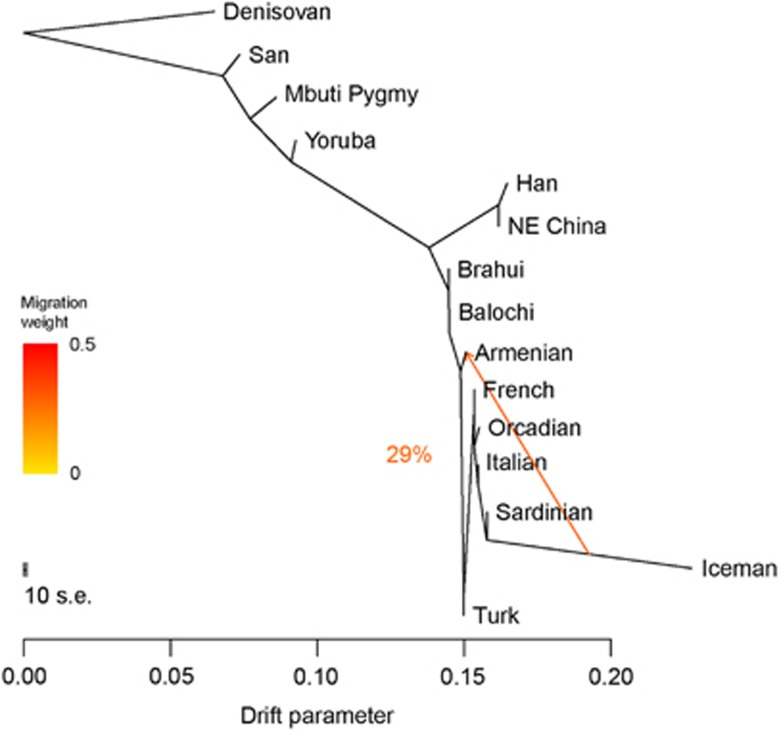
Inferred population tree with one mixture event. The graph was inferred by *TreeMix* allowing one migration event. The migration arrow is coloured according to its weight; the weight is correlated with the ancestry fraction and shows that 29% of Armenian ancestry is derived from a population related to ancient Europeans. The graph is stable in 100 bootstrap replicates.

We merged our data set with the genome of the Tyrolean Iceman, a 5300-year-old individual discovered on the Italian part of the Ötztal Alps. We used *TreeMix*^19^ to construct a tree of genetic relationships using representative regional populations plus Armenians and Turks from the Near East. *TreeMix* uses a model that allows for both population splits and gene flow to better capture historical relationships between populations. We obtained a tree that recapitulates the known relationships among population groups. Furthermore, the tree shows that the Iceman shared drift with Sardinians, as previously reported.^21^ We then ran *TreeMix* allowing it to infer only one migration event, and revealed gene flow from the Iceman to Armenians, accounting for about 29% of their ancestry. The graph structure appeared robust in 100 bootstrap replicates with the first migration (highest weight and lowest *P*-value), always leading from the Iceman to Armenians (Figure 4).

This structure was further investigated using outgroup *f3* statistics.^3, 18^ The expected value of *f3(Yoruba; Iceman, X)* in the absence of admixture with Yoruba will be a function of the shared genetic history of the Iceman and *X* (non-African populations). Most shared ancestry of the Iceman is with Sardinians and other Europeans (Supplementary Figure S3). This is followed by shared ancestry with some Near Eastern populations: Cypriots, Sephardic Jews, Armenians, and Lebanese Christians. Other Near Easterners such as Turks, Syrians, and Palestinians show less shared ancestry with the Iceman.

To investigate if the affinity of the Near East genetic isolates to Europeans preceded the arrival of the early farmers to Europe (represented by the Iceman), we repeated the outgroup *f3* statistics and replaced the Iceman with a 7000-year-old European hunter-gatherer from Spain (La Braña).^24^ West European hunter-gatherers have previously been shown to have contributed ancestry to all Europeans but not to Near Easterners.^6^ Consistent with this, we found reduced affinity and no noticeable structure in the Near Easterners in their relation to La Braña (Supplementary Figure S4; compared with the Iceman).

## Discussion

The origins of the Armenians and their cultural uniqueness are poorly understood. Here, we investigate the information that can be obtained by genetic analysis of present-day Armenians and comparisons with other present-day and ancient samples.

The position of the Armenians within global genetic diversity appears to mirror the geographical location of Turkey, which forms a bridge connecting Europe, the Near East, and the Caucasus. Turkey's location and history have placed it at the centre of several modern human expansions in Eurasia: it has been inhabited continuously since at least the early Upper Palaeolithic,^26^ and has the oldest known monumental complex built by hunter-gatherers in the tenth millennium bce.^27^ It is believed to have been the origin and/or route for migrating Near Eastern farmers towards Europe during the Neolithic,^28^ and has probably also played a major role in the dispersal of the Indo-European languages.^29^

We investigated Armenians further by inferring their admixture history. The Armenians show signatures of an origin from a mixture of diverse populations occurring from 3000 to 2000 bce. This period spans the Bronze Age, characterized by extensive use of metals in farming tools, chariots, and weapons, accompanied by development of the earliest writing systems and the establishment of trade routes and commerce. Many civilizations such as in ancient Egypt, Mesopotamia, and the Indus valley grew to prominence. Major population expansions followed, triggered by advances in transportation technology and the pursuit of resources. Our admixture tests show that Armenian genomes carry signals of an extensive population mixture during this period. We note that these mixture dates also coincide with the legendary establishment of Armenia in 2492 bce. Admixture signals decrease to insignificant levels after 1200 bce, a time when Bronze Age civilizations in the Eastern Mediterranean world suddenly collapsed, with major cities being destroyed or abandoned and most trade routes disrupted. This appears to have caused Armenians' isolation from their surroundings, subsequently sustained by the cultural/linguistic/religious distinctiveness that persists until today. The genetic landscape in most of the Middle East appears to have been continuously changing since then. For example, we detect East Asian ancestry in Turks from admixture occurring 800 (±170) years ago coinciding with the arrival of the Seljuk Turks to Anatolia from their homelands near the Aral sea. We also detect sub-Saharan African gene flow 850 (±85) years ago in Syrians, Palestinians, and Jordanians consistent with previous reports of recent gene flow from Africans to Levantine populations after the Arab expansions.^5, 30^

The admixture pattern in Armenians appears similar to patterns we have observed in some other genetic isolates in the region, such as Sephardic Jews and Lebanese Christians, who show limited admixture with culturally different neighbouring populations in the last two millennia.^5^ Our tests suggest that Armenians had no significant mixture with other populations in their recent history and have thus been genetically isolated since the end of the Bronze Age, 3000 years ago. In recent times, we detect genetic structure within the Armenian population that developed ~500 years ago. The date coincides with the start of the Ottoman–Persian wars and the split of Armenia into West and East between the Ottoman Empire in Turkey and the Safavid Empire in Iran.

One of the most-studied demographic processes in population genetics is the Neolithic expansion of Near Eastern farmers into Europe beginning ~8000 years ago. Armenians' location at the northern tip of the Near East suggests a possible relationship to the expanding Neolithic farmers. We find in Armenians and other genetic isolates in the Near East high shared ancestry with ancient European farmers, with ancestry proportions being similar to present-day Europeans but not to present-day Near Easterners. These results suggest that genetic isolates in the Near East – Cypriots (an island population), Near Eastern Jews and Christians (religious isolates), and Armenians (Ethno-linguistic isolate) – probably retain the features of an ancient genetic landscape in the Near East that had more affinity to Europe than the present populations do. Our tests show that most of the Near East genetic isolates' ancestry that is shared with Europeans can be attributed to expansion after the Neolithic period.

Armenians' adoption of a distinctive culture early in their history resulted in their genetic isolation from their surroundings. Their genetic resemblance today to other genetic isolates in the Near East, but not to most other Near Easterners, suggests that recent admixture has changed the genetic landscape in most populations in the region. Armenians' genetic diversity reveals that the ancient Near East had higher affinity to Neolithic Europe than it does now, and that Bronze Age demographic processes had a major impact on the genetics of populations in this region.

The importance of populations like the Armenians is not limited to the study of past demographic processes; isolated populations are emerging as a powerful tool for many different genetic investigations such as rare variant associations with complex phenotypes and the characterization of gene–environment interactions.^31^ Armenians' emergence from founders in the Bronze Age, accompanied by a long period of subsequent isolation, may have enriched rare disease alleles and therefore merits future medical exploration.

## Figures and Tables

**Table 1:** Source populations and admixture time for Armenians

*Source 1*	*Source 2*	f3*-statistics*^a^	z*-score*	*Time*±*SE*	P*-value*
West Europeans	Sub-Saharan Africans	–0.00105083	–5.01009	5826.52±672.84	2.00E–08
Caucasus populations	Sardinians	–0.000251561	–4.89462	5554.64±361.76	4.10E–36
Central and South Asians	Sardinians	–0.00110183	–16.261	4886.28±271.32	3.00E–34
Caucasus populations	Arabian Peninsula populations	–0.000467324	–7.32053	4812.08±381.64	8.10E–22
Caucasus populations	North Levantines	–0.000237086	–5.99602	4673.2±343	9.10E–19
South Levantines	Caucasus populations	–0.000235109	–5.03679	4376.12±426.16	7.60E–19
Central and South Asians	West Europeans	–0.000345672	–5.42631	4233.04±1111.6	0.0029
North Europeans	Arabian Peninsula populations	–0.000433178	–5.1784	3939.88±239.96	1.70E–32
Central and South Asians	North Levantines	–0.000484051	–6.95695	3908.52±682.08	2.10E–07

a Lowest *f3* resulted from each source region.
