# Supplementary figures and images for: Genetic evidence for an origin of the Armenians from Bronze Age mixing of multiple populations
Source: Eur J Hum Genet. 2015 Oct 21;24(6):931–6. doi: 10.1038/ejhg.2015.206 (PMC4820045; doi:10.1038/ejhg.2015.206)

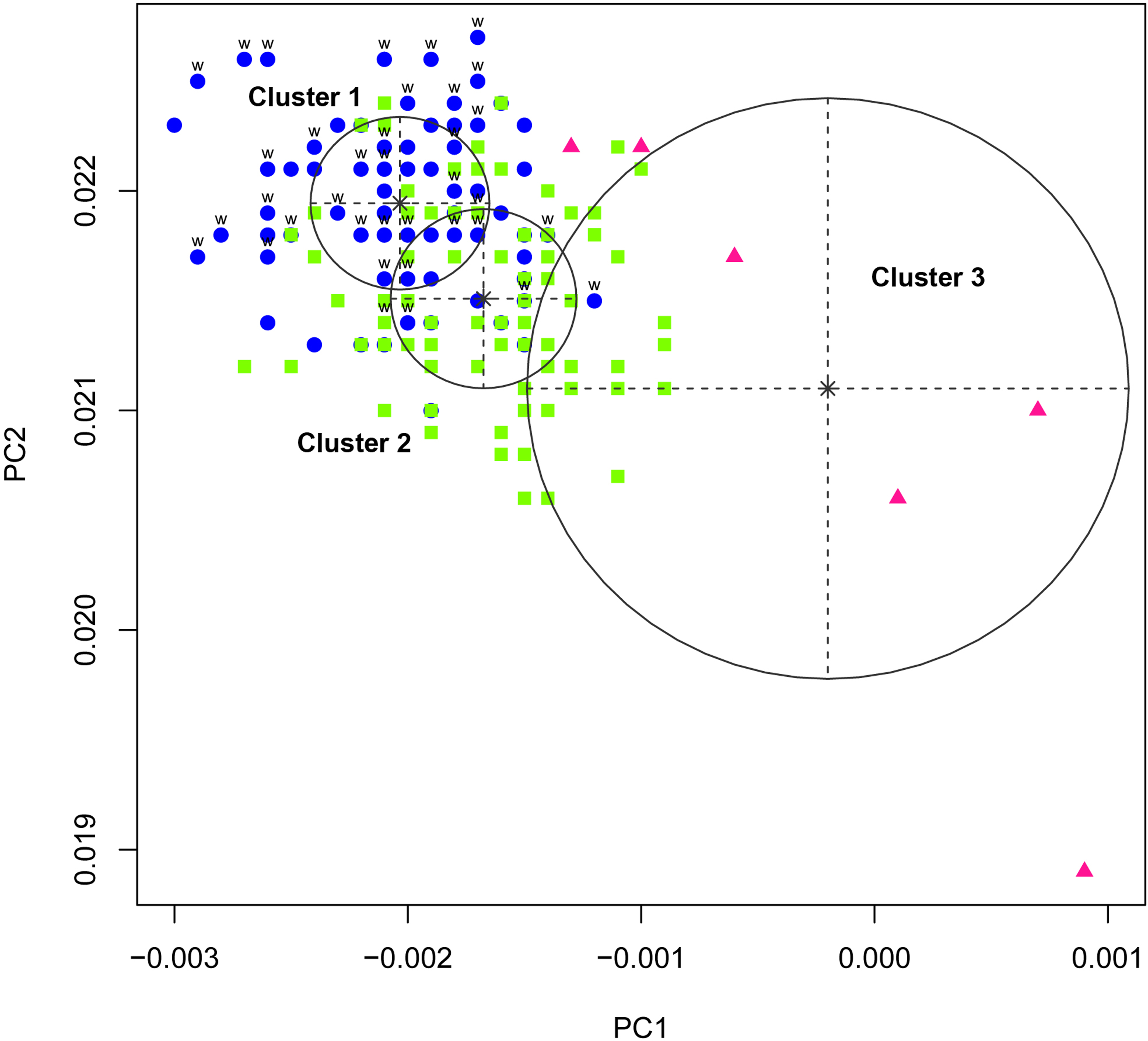

Supplement: Supplementary Figure 1 [file ejhg2015206x2.tif]

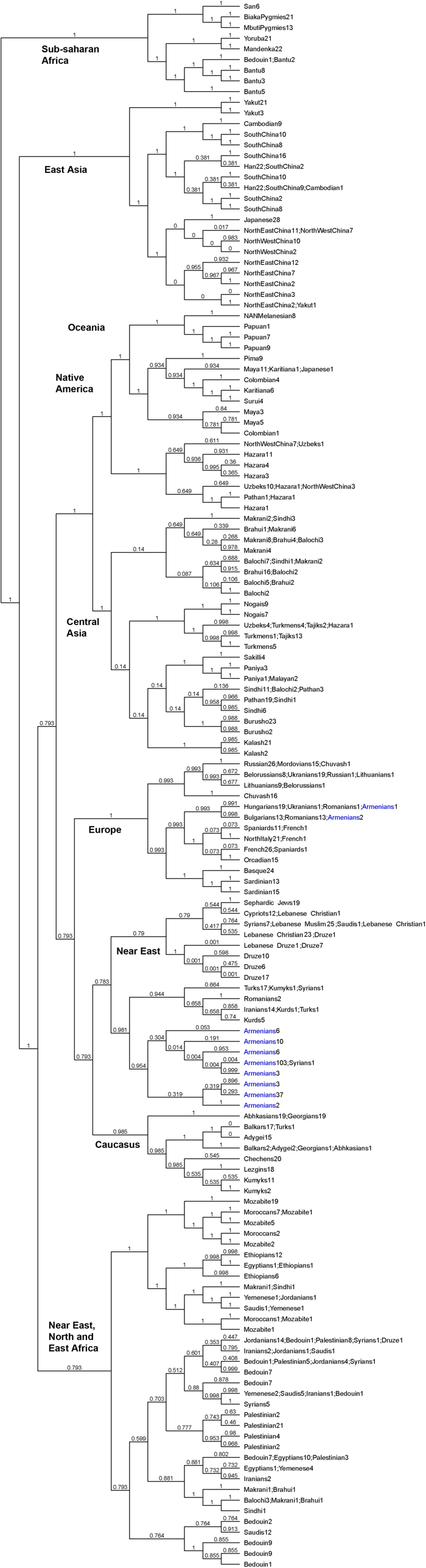

Supplement: Supplementary Figure 2 [file ejhg2015206x3.tif]

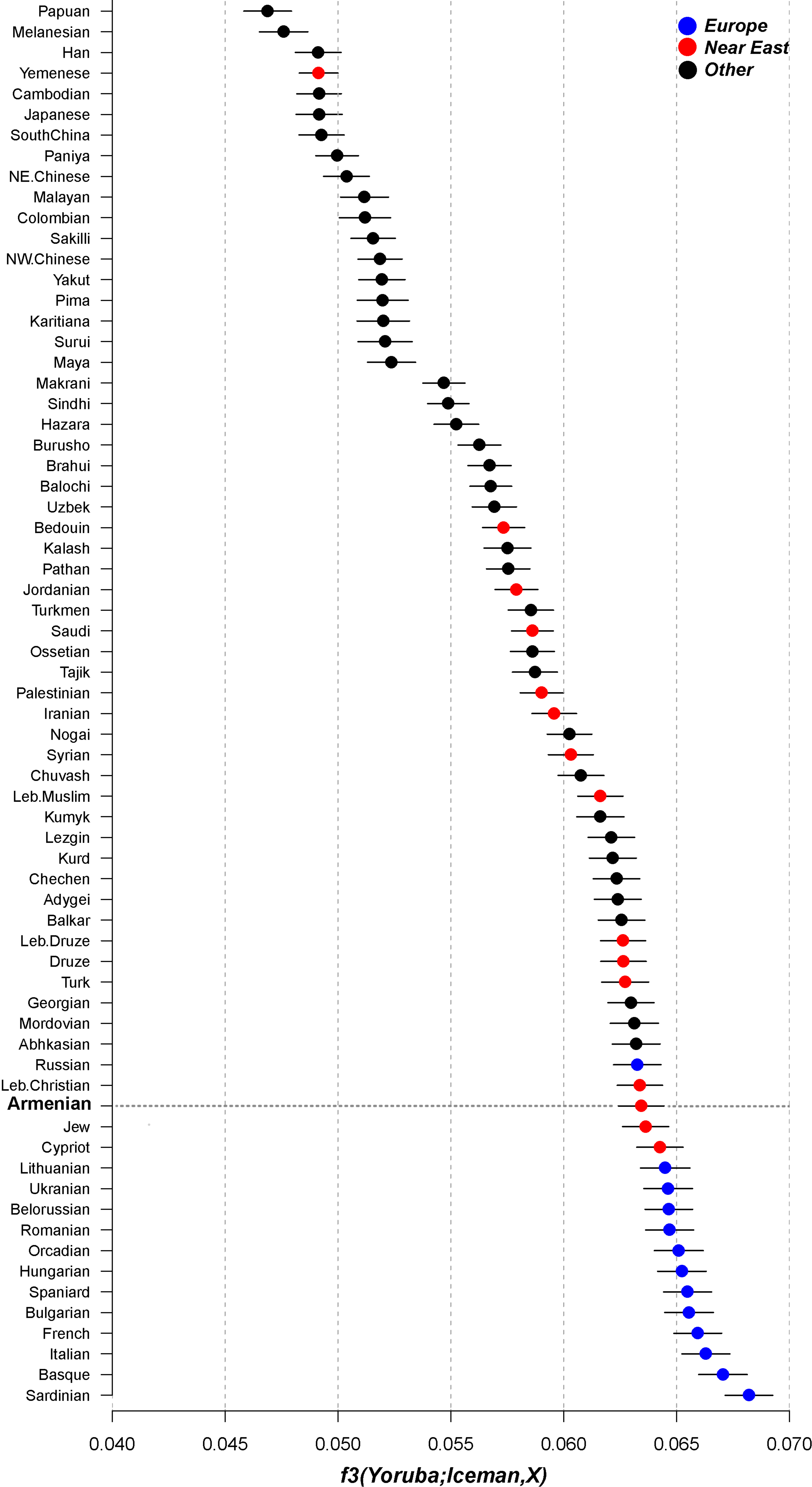

Supplement: Supplementary Figure 3 [file ejhg2015206x4.tif]

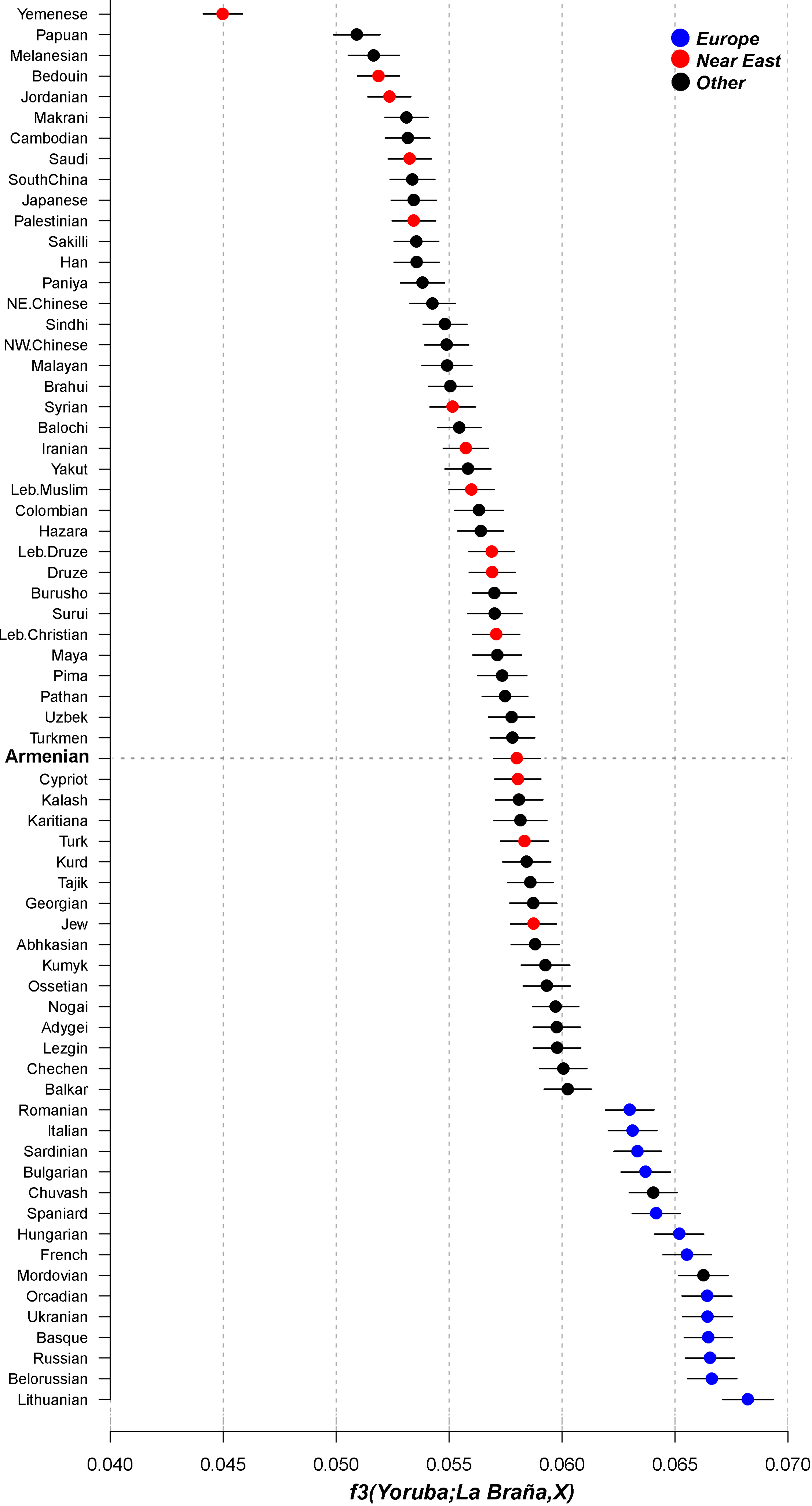

Supplement: Supplementary Figure 4 [file ejhg2015206x5.tif]
