# Supplementary material for: Genetic evidence for an origin of the Armenians from Bronze Age mixing of multiple populations
Source: Eur J Hum Genet. 2015 Oct 21;24(6):931–6. doi: 10.1038/ejhg.2015.206 (PMC4820045; doi:10.1038/ejhg.2015.206)
Supplement: Supplementary Information [file ejhg2015206x6.doc]

**Supplementary info**

**Figure S1.** **Genetic structure in Armenians.** *MCLUST* classifies Armenians into three clusters. Cluster 1 (blue) includes 95% of the Armenians that trace their origin to Western Armenia (East Turkey) (labelled W). Cluster 1 also includes 33% of the general Armenians (recruited from modern Armenia). Cluster 2 includes 57% of the general Armenians. Cluster 3 includes six Armenians recruited from Chambarak (modern-day Armenia) or Maykop (Republic of Adygea, Russia).

**Figure S2. Population relationships from genome-wide haplotypes**. Each tip of the tree corresponds to an individual; numbers of individuals are shown next to their population name at the tip of the branches. Numbers on branches show partition posterior probability. Armenians are shown in blue, forming two major clusters in a Near Eastern branch.

**Figure S3. Shared genetic drift between worldwide populations and the Tylorean Iceman, a 5,300 year old European.**

**Figure S4. Shared genetic drift between worldwide populations and La Braña, a 7,000 year old European.**
